# Key considerations based on pharmacokinetic/pharmacodynamic in the design of antibody-drug conjugates

**DOI:** 10.3389/fonc.2024.1459368

**Published:** 2025-01-09

**Authors:** Yangyang Gao, Yuwei Xia, Yixin Chen, Shiqi Zhou, Yingying Fang, Jieru Yu, Leyin Zhang, Leitao Sun

**Affiliations:** ^1^ The First Affiliated Hospital of Zhejiang Chinese Medical University (Zhejiang Provincial Hospital of Chinese Medicine), Hangzhou, Zhejiang, China; ^2^ College of Basic Medical Science, Zhejiang Chinese Medical University, Hangzhou, China; ^3^ Department of Oncology, Hangzhou TCM Hospital of Zhejiang Chinese Medical University (Hangzhou Hospital of Chinese Medicine), Hangzhou, China; ^4^ Academy of Chinese Medical Science, Zhejiang Chinese Medical University, Hangzhou, China; ^5^ Key Laboratory of Neuropharmacology and Translational Medicine of Zhejiang Province, School of Pharmaceutical Sciences, Zhejiang Chinese Medical University, Hangzhou, China

**Keywords:** antibody-drug conjugate, pharmacokinetic/pharmacodynamic, targeted antibodies, toxins, pharmacokinetics, pharmacodynamics

## Abstract

**Background:**

Antibody-drug conjugate (ADC) is an anticancer drug that links toxins to specifically targeted antibodies via linkers, offering the advantages of high target specificity and high cytotoxicity. However, complexity of its structural composition poses a greater difficulty for drug design studies.

**Objectives:**

Pharmacokinetic/pharmacodynamic (PK/PD) based consideration of ADCs has increasingly become a hot research topic for optimal drug design in recent years, providing possible ideas for obtaining ADCs with desirable properties.

**Methods:**

From the assessment of the ADC action process based on PK/PD, we introduce the main research strategies of ADCs. In addition, we investigated the strategies to solve the prominent problems of ADC in the clinic in recent years, and summarized and evaluated the specific ways to optimize various problems of ADC based on the PK/PD model from two perspectives of optimizing the structure and properties of the drugs themselves. Through the selection of target antigen, the optimization of the linker, the optimization of novel small molecule toxins as payload, the optimization of ADC, overcoming the multi-drug resistance of ADC, improving the ADC tumor penetration of ADC, surface modification of ADC and surface bystander effect of ADC provide a more comprehensive and accurate framework for designing new ADCs.

**Results:**

We’ve expounded comprehensively on applying pharmacokinetics or pharmacodynamics while designing ADC to obtain higher efficacy and fewer side effects. From the ADC’s PK/PD property while coming into play *in vivo* and the PK/PD study strategy, to specific ADC optimization methods and recommendations based on PK/PD, it has been study-approved that the PK/PD properties exert a subtle role in the development of ADC, whether in preclinical trials or clinical promotion.

**Conclusion:**

The study of PK/PD unfolds the detailed mechanism of ADC action, making it easier to control related parameters in the process of designing ADC, limited efficacy and inevitable off-target toxicity remain a challenging bottleneck.

## 1 Introduction

Antibody-drug conjugate (ADC) is a hot therapeutic agent for the treatment of various malignancies. These drugs consist of a biologically active cytotoxic payload, a chimeric monoclonal or polyclonal antibody (1), and a binding linker. The antibody targets cell surface antigens specifically expressed by certain tumor cells, then the cytotoxic payload is released from the ADC after internalization and cleavage of the linker, ultimately triggering the death of the tumor cell (2, 3). With their high-targeted specificity and high cytotoxicity advantages, ADCs are becoming a hot topic in medical research (4). Nowadays, ADCs are not only used to treat hematological malignancies but have been approved to treat many solid tumors by targeting specific antigens. For example, Trastuzumab Emtansine (T-DM1) and Trastuzumab Deruxtecan (T-DXd) are approved for the treatment of HER2-positive breast cancer and gastric cancer by the Food and Drug Administration (FDA). In this sense, ADCs that are approved currently can be regarded as targeted chemotherapy (5).

However, due to its complex, diverse structure and the low content of small molecular toxins released in the circulatory system, inevitable issues on the safety and invalidity aspects in the current ADC drug design (6), such as the inefficiency of antibody recognition and localization of tumor cells, the instability of linkers in the transport process, and the residue of small molecular toxins in the body. Due to the complexity of the molecular structure of ADCs, different kinds of ADC molecules may have great differences. Even for ADC drugs acting on the same target, the antigenic epitopes, linkers, and small toxins of different molecules are not completely the same (7). In the development and clinical application of antibody-drug conjugates (ADCs), pharmacokinetics (PK) and pharmacodynamics (PD) offer critical insights into understanding *in vivo* drug processes and their mechanisms of action. For example, it was reported by Sukumaran and colleagues that a platform model can predict the complex PK behavior of ADCs with protease-cleavable valine-citrulline (VC) linker by incorporating known mechanisms of ADC disposition (8). Nevertheless, a series of challenges persist in their implementation. Consequently, it is still necessary to concentrate on key components such as antibodies, ligands and small molecular toxins to optimize the combination strategy of ADC design.

## 2 Assessment of ADC action process based on PK/PD

### 2.1 Pharmacokinetic

Absorption, distribution, metabolism, and excretion (ADME) constitute the four essential processes through which ADC functions *in vital*. These processes are pivotal considerations in the comprehensive study of drugs based on PK (9). Endocytosis and intracellular transport refer to the continuous process in which cells take molecules fromintestine outside the cell and internalize them into the cytoplasm, where they are then broken down through complex enzymatic pathways, which is the pivotal step for ADC to function (10). Exploring ADME, endocytosis and intracellular are imperative for a thorough understanding of the entire *in vivo* action sequence of ADC and is equally crucial for refining ADC design details to enhance clinical efficacy.

#### 2.1.1 Absorption

Absorption represents the initial stage in the ADME process and signifies the commencement of investigating the PK of ADC. Given that ADC is a protein-based pharmaceutical, it is primarily administered via intravenous injection in clinical settings (11). Subsequent to intravenous administration, ADCs can permeate the interstitial matrix either through convective plasma flow across vascular pores or capillary filtration (12). To achieve optimal efficacy, dosing of ADC typically aims to attain maximum systemic target occupancy.

#### 2.1.2 Distribution

After intravenous administration, ADCs are progressively distributed to target sites in various body tissues along the systemic blood circulation. The half-life and plasma clearance rate during the distribution process of ADCs constitute a crucial aspect of PK studies. Antibodies with low clearance rates and long half-life are undoubtedly a good choice for ADC design and these characteristics also provide convenience in reducing the frequency of administration (13). Compared with the payload component, the antibody of ADCs are the primary driver of the slow clearance, long systemic half-life and restricted tissue distribution of these modalities (14). Fc receptors bind to the Fc region of antibodies and help regulate their distribution and elimination in the body. The Fc receptors that interact with IgG-based drugs are the neonatal Fc receptor (FcRn) and the FC-γ receptor family (FcγR). It has been shown that in endothelial cells, most of the FcRn-bound ADCs are returned to the plasma, while a small amount is excreted into the interstitial fluid on the basolateral surface (12). This cycle is beneficial for the prolonged plasma half-life of ADC, which can help further the binding of ADCs to target tumor cells, thereby improving the efficacy of ADC. Moreover, it has been demonstrated that as a macromolecular entity, ADCs exhibit enhanced extravasation from kidney, bone marrow, or lymphoid organs toward leaky tumor vessels and sinus vessels due to the enhanced permeability and retention (EPR) effect (15). The EPR effect denotes the phenomenon wherein certain macromolecular drugs more readily penetrate into tumor tissues and persist for extended durations compared to normal tissues. Consequently, this facilitates ADC utilization within tumor tissues while mitigating the toxicity of ADC dissemination to normal tissues—a highly advantageous feature for solid tumor treatment (16), thereby enhancing PK.

In addition, the distribution of ADC in the target area varies depending on the tissue, and a comprehensive study of this process can help to use ADC’s characteristics to avoid many administration risks and help the drug to reach the target position better. Because the structure of ADCs is similar to that of antibodies, the distribution of ADCs is similar to that of antibodies in the body and is also affected by many of the same physiological processes in the body. ADCs are mainly distributed in the skin, lungs, liver, kidneys, and other tissues (17). Its numbers in different tissues are influenced by target binding and physiochemical properties (14). After sufficient time in circulation, ideally, most ADCs will eventually be distributed near the tumor tissue (16). Nevertheless, ADC, as an exogenous biomolecule, may stimulate humoral immunity after entering the body, causing anti-ADC immune responses, accelerating the inactivation or clearance of ADC, and hindering its tissue distribution (18). For example, the hydrophobicity of payloads will likely lead to the aggregation of ADCs, which in turn increases their overall immunogenicity and affect the distribution of ADC. As the core component of ADC, the choice of antibodies is one of the key factors leading to immune responses. Although most ADCs currently use humanized or fully human monoclonal IgG antibodies as their backbone, the hapten-like structure of ADC theoretically means that it carries a higher risk of inducing an immune response than traditional monoclonal antibody therapies (19), and different subtypes of IgG antibodies have different effects on the immune system (20). The blood drug concentration of ADCs is also one of the factors affecting the distribution. Excess ADC concentration can be more widely distributed around the tumor tissue to saturate tumor surface targets (21). If the dose is insufficient, ADCs will be mainly distributed near tissues that have high expression of the target or are highly perfused and permeable (12, 22). Furthermore, when the drug leaks out, it will concentrate first in the proximity of the solid tumor to the vascular portion and rapidly bind to the highly expressed cellular target antigenic sites, while the interior is difficult to access the monoclonal antibody (mAb), so a typical perivascular tumor distribution is formed (23). The result is that the distribution of mAb within solid tumors exhibits a high degree of concentration heterogeneity and the actual exposure of ADC to tumors is low. This may lead to supersaturation toxicity of ADC to fractions close to blood vessels and lack of expected effect on fractions away from blood vessels, thus reducing the efficacy and existence time of ADC presence *in vivo* (24, 25).

#### 2.1.3 Endocytosis and intracellular

Endocytosis can be divided into receptor-dependent (clathrin-mediated or caveolae-mediated) and receptor-independent (clathrin-caveolin-independent endocytosis) categories (26). The antibody on the ADC specifically binds to the tumor surface antigen to pass through the membrane barrier into the cell. Then, the acidity of ADC early endosomes is increased by V-ATPase activity and is gradually converted to late endosomes by fusion with homotypic endosomes into larger vesicles (27). The late endosome then fuses with the lysosome, causing a drop in pH and triggering the dissociation of the linker, releasing the drug to reach the targeted area, leading to a disruption of tubules or cell cycle arrest, ultimately causing cancer cell apoptosis (10). The endocytosis efficiency of ADCs is related to the endocytic properties of the antigen, the antibody binding site on the antigen, and the tumor cell type (28). The payload can be delivered intracellularly by the internalization of the antibody in conjunction with cellular ligand, so the endocytic properties of the target is a key determinant of the selection of the appropriate antigen (28). Because of the different endocytic properties of the antigen, modifying antibody structure can improve endocytosis efficiency, such as Bispecific antibodies (those that bind two targets) (29). When bispecific antibodies are used, ADC can simultaneously bind to two target antigens on a target tumor cell. The antigen with high endocytosis efficiency is responsible for internalizing the ADC to improve endocytosis efficiency (30). Internalization is important for optimizing the dosing regimen to maximize the therapeutic index and the improvement of endocytosis efficiency contributes to the improvement of curative effect.

#### 2.1.4 Metabolism and excretion

ADCs are eliminated through metabolism and excretion, which is the final step of ADME. After being broken down and functioning, ADCs that have become small molecule drugs and fragments are finally released by tumor cells, which may produce bystander effect (31). This effect can cause toxic drugs to further destroy neighboring tumor cells and enhance the curative effect, but the metabolites of ADC should be excreted in time to prevent unnecessary immune reactions or damage to the liver and kidneys, which are the two major organs for drug clearance (11). Many factors related to clearance, such as clearance rate and accumulation after multiple administrations, are the main research directions of ADC PK/PD. The mechanisms of drug clearance are mainly divided into three categories: metabolic transformation, biliary excretion and renal excretion (32).

**Figure 1:**
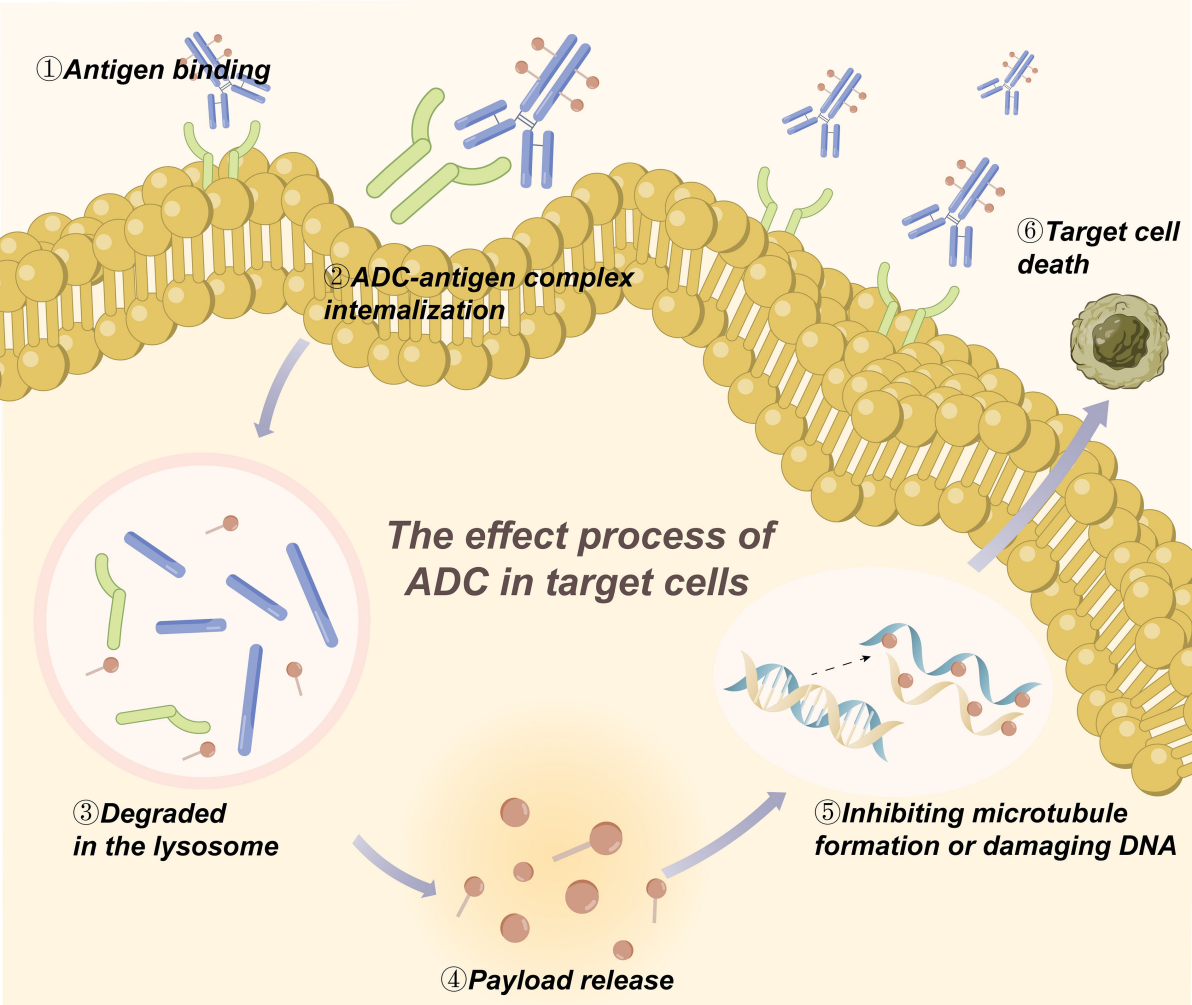
The metabolism of ADC in the target tumor cells.

Liver is the main metabolic organ of drugs, and liver cells contain a large number of uptake/efflux transporters and abundant enzymes for drug metabolism, such as phase I metabolizing enzymes and cytochrome P450 enzymes. Most small molecules are either metabolized by the liver through phase I and/or phase II reactions or excreted in whole or in part from the kidneys (32).In general, the metabolism of ADC occurs mainly inside the target tumor cells, which is the key step for ADC to exert its actual effect and is the focus of PK/PD research. Ideally, ADCs bind to target antigens on the surface of tumor cells and enter the tumor cells through intracellular chemotaxis. And then ADCs are transported to the intracellular lysosome, where they are hydrolyzed by the corresponding enzymes to break the linkers and release the cytotoxic payload into the cytoplasm. In the cells, the catabolite or linker-payload fragment formed after the degradation of linkers must retain its cytotoxic activity to play subsequent roles (12). Most of the payloads act on tumor cells by inhibiting microtubule formation or damaging DNA and can damage neighboring cells through bystander effects (**Figure 1**) (31). As the cytotoxic payloads are released, the drug-antibody ratio (DAR) value decreases until it becomes 0 (33). Hence, the rate of release of cytotoxic payloads can be understood by measuring the rate of change of DAR in blood, and the metabolic process of ADC can be investigated comprehensively (16). Besides serving the intended purpose, the payload also has the potential to elicit an immune response. The two main mechanisms of payloads to initiate immune responses against tumor cells and play a therapeutic role are direct stimulation of adaptive immunity and indirect immunogenic cell death (ICD). In ICD, to cause tumor cell death, payloads induce the release of antigenic molecules from cells in a specific manner, which triggers immune responses of tumor-infiltrating immune cells (21). It has been shown that some payloads can induce strong immune responses against the target tumor, and this mechanism would potentially be another way for ADCs to work. For example, the recruitment of immune cells occurs after T-DM1 treatment of HER2(+) tumors (34).

The kidney is the main excretory organ of drugs and their metabolites. Renal excretion consists of three main processes, namely, glomerular filtration, active renal tubule secretion and tubular reabsorption. Free small molecule drugs that are not bound to plasma proteins enter proximal renal tubules through glomerular filtration, while ADCs, antibodies, larger molecular weight peptides and amino acid fragments are not excreted by glomerular filtration and are reabsorbed and reused as amino acids (35). In addition, small molecule metabolites can be eliminated by enzymatic metabolism and excreted in the feces via transporter proteins with bile from the biliary tract to the intestine (11). Thus, for drugs with low clearance rates, appropriate strategies should also be taken to improve their clearance rate *in vivo* to prevent accumulated toxicity. The success rate of drug development can be effectively improved by improving the clearance rate to achieve the best balance of safety, activity and pharmacokinetic properties.

### 2.2 Pharmacodynamic

Like all other drugs, ADCs have a duality in their course of action, i.e., therapeutic effects versus adverse effects, which is the core of PD studies. The results of numerous clinical trials have shown that despite the encouraging efficacy of ADCs in poor prognosis and refractory tumors such as mutant lung cancers (36), triple-negative breast cancers (37), and malignant lymphomas (38), the adverse effects caused by off-target toxicity cannot be ignored. Off-target toxicity, i.e., the damage of normal tissue cells caused by ADCs due to poor linker stability breaking early to release the payload and non-specific distribution of the payload during transport to target cells or target tissues (39). This is generally caused by non-optimal engineering of the drug (37), it is critical to reduce off-target toxicity by optimizing the ADC structure design. It has been found that at least part of the off-target toxicity is caused by elevated levels of G0F in the mAb portion of the ADC composition.G0F is an agalactosylated glycan on the Fc fragment of the antibody. The absence of its terminal galactose significantly increases the likelihood of mAb interaction with cell surface mannose receptors (MR), thereby increasing the risk of ADC off-targeting (40). Additionally, the aggregation effect of ADCs with FcγR-activating properties is significantly enhanced in cells, also exacerbating the potential for off-target toxicity. Therefore, modifying Fc to silence FC-mediated effector function or using FC-γR to block antibodies may be effective measures to reduce off-target toxicity (41).

Currently, ARX788, developed by Lillian Skidmore et al. for the treatment of drug-resistant breast and gastric cancers uses a stable oxime bond, along with a non-cleavable drug linker to enable specific attachment of the payload to the antibody in conjunction (42). As well as 9MW2821, developed by Zhou et al. for targeting nectin-4 using a thioether bridge linker with a high degree of cyclic stabilization, both of which have demonstrated strengths in clinical trials in terms of reducing off-target toxicity and enhancing drug safety (43).

## 3 Specific strategies to optimize ADC based on PK/PD

**Figure 2:**
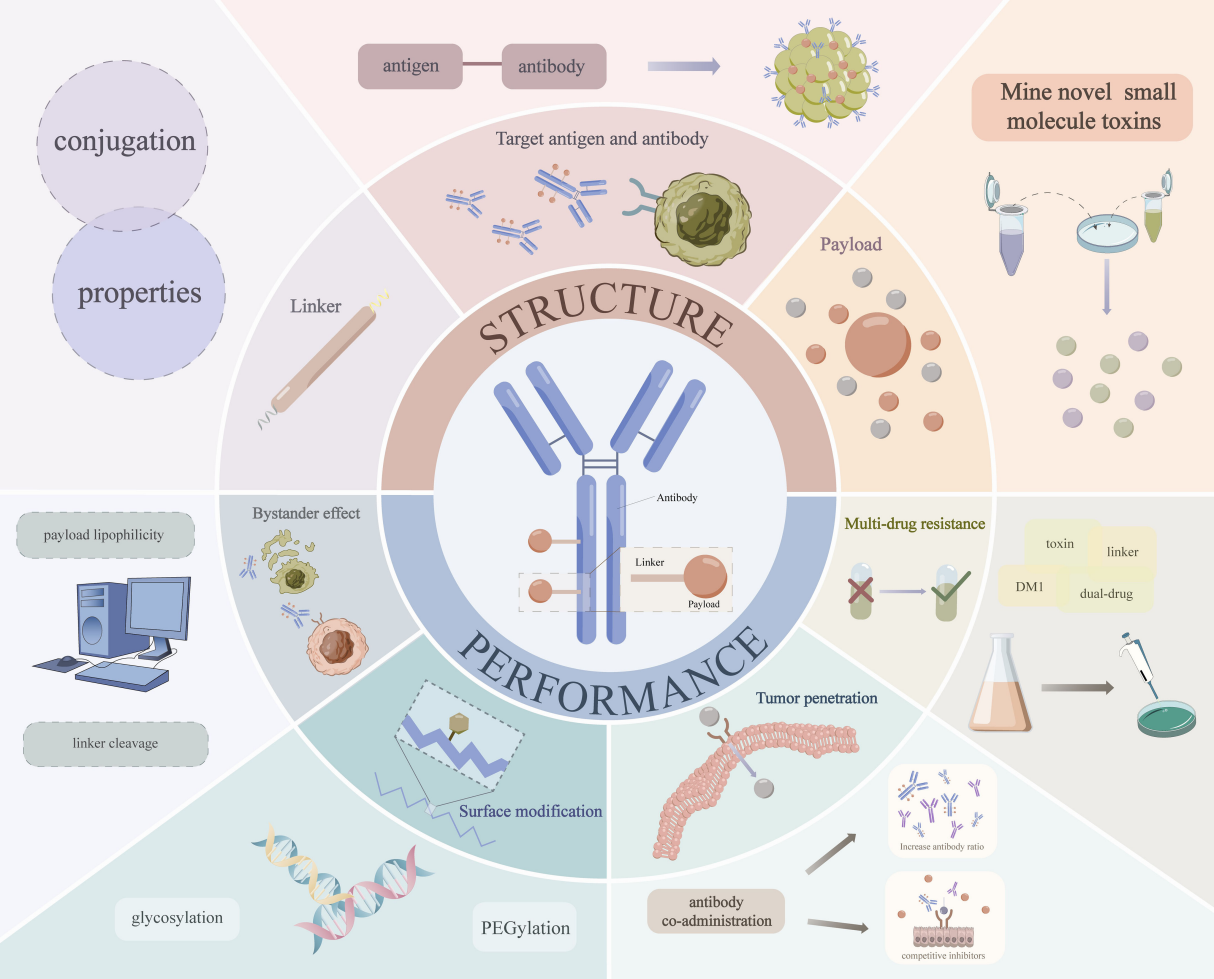
Specific strategies to optimize ADC based on PK/PD.

However, due to the emergence of drug resistance (44) and the instability of the linker (45), etc., the concentration of cytotoxic drugs delivered to intracellular targets through ADCs is still very low (46), resulting in less than ideal efficacy in tumor therapy. Previous studies have shown that designing ADCs based on PK/PD can help optimize drug performance and ultimately facilitate their successful preclinical-to-clinical translation (44, 47). Based on these we investigated the strategies to solve the prominent problems of ADCs in the clinic in recent years, and summarized and evaluated the specific ways to optimize various problems of ADC based on the PK/PD from two perspectives of optimizing the structure and properties of the drugs themselves (**Figure 2**).

### 3.1 Structure optimization

#### 3.1.1 Target antigen and antibody

The first thing to consider in the design of optimizing ADCs based on the PK/PD is the selection of the target antigen. To make the ADC as effective as possible on the target cells, the target antigen should be uniformly distributed and highly expressed on the surface of the target cells, while not expressed or lowly expressed on the surface of normal tissues. And it should have low shedding, to avoid the antigen shed from the target cells to bind a large number of conjugated drugs in the blood circulation, which leads to the actual concentration of drugs cannot reach the therapeutic requirements (16, 48). It has also been reported that antigens expressed in the tumor microenvironment (TME) are more readily captured by ADC in the circulating blood, and therefore targeting antigens in the TME can achieve better anti-tumor effects (49). Additionally, some target cell surface antigens have more significant advantages than others, for example, targeting VEGFR-1 may represent a multi-targeted therapeutic option. As VEGFR-1 is expressed on the surface of tumor cells, tumor-associated vascular endothelium, and pro-tumoral myeloid cells. ADC designed to target this antigen has the triple effect of directly killing tumor cells, inhibiting angiogenesis, and tumor infiltration by immunosuppressive immune cells (50).

Antibodies are the core components of ADC, so optimizing drug design from the perspective of inherent nature and structure is one of the important strategies to enhance the drug’s specificity to reach the target cells. Early ADC design mostly used mAbs as a drug component, which have high binding affinity (16). While subsequent studies have shown that when mAb exudes, they often form typical perivascular tumor distribution (23), which causes uneven distribution of antibodies. It is evident that only determining the optimal value of antibody binding affinity is a feasible way to optimize antibodies. Most ADCs currently control binding affinity in the range of 0.1 to 1.0 nM, but there is a lack of studies describing the optimal binding affinity index of antibodies (16). In addition, from the structural perspective, reducing immunogenicity in humans by chimeric humanized antibodies and using antibodies with a long half-life and high molecular weights can also be optimized (48), to prevent antibody clearance and increase effective antibody concentration.

The development of novel antibodies is the direction to optimize the overall PK/PK properties of ADC in recent years. For example, Hu and Zhou et al. proposed LR004 as an anti-EGFR antibody with significant application in the treatment of malignant tumors (51, 52). LR004 has a more human-like sialic acid and its glycoengineering modification structure is less immunoreactive, giving it a longer serum half-life and higher thermostability. Furthermore, their experimental results showed that LR004 has high binding affinity and internalization ability as an ADC component, and exhibited stable safety and desirable PK in mouse models (52). Kang and his colleagues developed HER2-based specific pertuzumab antibody-drug conjugates by molecular design. Under different pH conditions (acidic vs. basic), the difference in affinity was up to 250-fold due to differences in the amino acid action of the molecular chains. Thus, they exhibit distinct binding potencies inside and outside target cells. This allows the antibodies to bind strongly extracellularly, improving the drug’s ability and stability to capture target cells. Then, when the antibodies are internalized, they bind less intracellularly, which enables the ADC to exert its effect rapidly. Results from HER2 xenograft tumor models in mice suggest that this engineered pertuzumab variant-designed ADC exhibits superior therapeutic efficacy compared to clinically-approved HER2-specific ADC (53). In addition, antibody affinity, epitope, and valence can be optimized by protein engineering of the antibody to improve the overall stability and immunogenicity of ADC (54). The antibody can also be improved to reduce its binding to non-target cells by using antibody fragments (fab, scFvs, vhs) or low-affinity monoclonal antibodies to improve monoclonal antibody penetration (25).

#### 3.1.2 Linker

The linker can be optimized in terms of two main parameters: the conjugation method and its self-properties. The first step is to choose the appropriate conjugation method. Chemical conjugation and enzymatic conjugation are currently the most widely used methods for tethering the antibody and payload components. Two of the conventional chemical conjugation methods (Lysine coupling and Cysteine coupling) have been FDA-approved and clinically tested, but may result in higher antibody clearance rates and lower antigen binding potency due to their significant DAR heterogeneity issues. Enzyme conjugation can achieve DAR homogeneity effectively, however, it has a more cumbersome process at the same time (N-glycan trimming, glycosylation, and conjugation) (45). The second is to optimize the properties of the linker itself based on PK/PD, including cleavability (45, 55), hydrophilicity (56), length, and steric hindrance (55), etc, to achieve the optimal solution of drug stability and linker cleavage efficiency.

Before ADCs reach tumor cells, what needs to be ensured is structural stability so that they can reach the anticipated location before they exert a therapeutic effect, preventing linker breakage and ADC metabolism before they bind to tumor cell surface antigens (16). Prematurely released cytotoxic payloads may be captured by non-target tissues or organs, especially when tissues with high blood flow and phagocytosis (e.g. liver, intestine) (57). The toxicity it causes should not be underestimated either (57). Linkers must be stable in plasma, interstitial fluid, and lymph to minimize off-target toxicity and ensure maximum delivery to the intracellular space of the tumor cells (12). According to their cleavage properties, linkers can be classified into cleavable linkers and non-cleavable linkers. Cleavable linkers need to require the proper cleavage efficiency, they can be designed to ensure whether ADC can be rapidly cleaved after inantigen with highternalization in target cells, releasing small molecule toxins for rapid therapeutic efficacy. While non-cleavable linker can be used to reduce off-target toxicity by circulating stably in the bloodstream upfront to target tumor antigen binding sites (45, 58).In general, non-cleavable linkers have longer half-lives and lower plasma clearance than cleavable linkers (59). However, when ADCs are absorbed into target tumor cells, cleavable linkers may be more advantageous to exert toxic effects quickly. Recent research has been focused on the development of more stable cleavable linkers, such as the cathepsin-responsive tripeptide linkers, and β-glucuronidase-cleavable linkers (60). We summarized the current main cleavable and non-cleavable linker, their main advantages and disadvantages, providing a reference for the selection of the linker in ADC design (**Table 1**).

**Table 1:** The advantages and disadvantages of various cleavable and non-cleavable linker at present described.

	Linker	Advantages	Disadvantages	References
Non-Cleavable linker	Thioether linker	Stabilization can result in efficacious ADCs	Unable to exert a bystander effect	(58, 61)
	Maleimide caproyl linker (e.g. Kadcyla)	Stabilization can result in efficacious ADCs	Unable to exert a bystander effect	(58, 61)
Cleavable linkers	Disulfide linker	Resisting reductive cutting in the cycle		(45, 58)
	Hydrazone linker		Gastrointestinal toxicity Low tolerability	(45)
	Pyrophosphate diester linker	Hydrophilicity Stability during circulation Traceless release of the payload	Unknown mechanism of lysosomal cleavage	(45)
	Cathepsin B-responsive linker (e.g. Val-Cit PABC and Val-Ala-PABC)			(45)
	β-Glucuronidase-cleavable linkers	Low level of aggregation High plasma stability		(58)
	β-Galactosidase-cleavable linkers	β-galactosidase exists only in lysosomes.		(58)
	Phosphatase-cleavable linkers	High blood stability Rapid lysosomal cleavage Aqueous solubility		(61)

The linker design also needs to take into account the hydrophilicity and hydrophobicity of the linker. DAR is an important attribute for measuring the drug specificity of ADCs, which is usually limited to 2-4. Studies have shown that by using hydrophilic linkers, it is possible to circumvent the accelerated plasma clearance *in vivo* due to high DAR and meanwhile satisfy the enhanced anti-tumor capacity brought by high DAR. Thus, homogeneous 8-loaded ADCs can be made without impairing pharmacokinetics (56). What can be seen from this is that improving the hydrophilicity of the linker may be an important strategy for ADC PK/PD-based optimization.

In addition, the design of a shorter linker often improves ADC stability by further binding the payload within the linker steric hindrance barrier of the antibody. However, linker steric hindrance is not always favorable, and there is also the possibility of slow or even ineffective payload release happening. Therefore, the determination of the optimal value still necessitates more precise consideration of trade-offs (55).

#### 3.1.3 Cytotoxic payload

The mining of novel small molecule toxins is a current research hot topic for payload-based optimization of ADC. It has been found that high levels of nitric oxide (NO) released by NO donors have surprising effects in the fight against cancer by virtue of their efficient tumor growth inhibitory effects, which can also be used as a cytotoxic small molecule toxin (62). However, the gas form of NO has limited its development in the previous years due to the difficulty of building stable NO donors as payload. Fortunately, in a study by Sun and colleagues, for the first time, HL-2 (with a disulfide bond and a maleimide terminus) was combined with an antibody that targets CD24 via a thioether bond to generate an ADC-like immunoconjugate, successfully creating a targeted NO donor mechanism. And the results of mouse experiments showed that HL-2 has efficient targeting with low side effects (63). Sun’s research is groundbreaking in the field of nitric oxide (NO) antitumor research and provides practical ideas for the development of cytotoxic payloads for antibody-drug conjugates (ADCs).

Pyrrolobenzodiazepine dimers (PBD) is a new type of highly cytotoxic small molecule toxin that blocks tumor cell proliferation by stopping the cell cycle at G2 to prevent cell division. And because its inhibitory effect is based on the premise of not changing the DNA helix, the DNA repair mechanism can be evaded. Thus, PBD can avoid the development of drug resistance and play an endurable and stable therapeutic effect (64). Alpha-Amanitin, a toxic bicyclic octapeptide, has received much attention in the field of ADC design research because of its highly selective inhibition of RNA polymerase II, small molecular weight, and good water solubility (65).

Additionally, we summarized the current main toxic payloads, their mechanisms for inhibiting tumor cells, as well as their main advantages and disadvantages, providing a reference for the selection of the payload in ADC design (**Table 2**).

**Table 2:** The inhibitory mechanism, advantages and disadvantages of the payloads at present described.

Payloads	Inhibitory mechanism	Advantages	Disadvantages	References
Auristatin (MMAE, MMAF)	Microtubule inhibitor	Stable High activity Strong immune specificity	Long-term remissions are rarely observed.	(66, 67)
Maytansinoids (DM1, DM4)	Microtubule inhibitor	Strong killing activity Good stability and solubility	Hard to conjugate, lack of selectivity for cancer cells	(16, 67)
Tubulysins	Microtubule polymerization inhibitor	Powerful antiproliferative activity		(16)
Halichondrin	Microtubule inhibitor	Unique effect on tumor microenvironment		(16)
Cryptomycins	Microtubule inhibitor		Hypertoxicity	(68)
Calicheamicin	DNA damaging agent	Strong killing effect Nonspecific damage effect on cell RNA		(16)
Pyrrolobenzodiazepine dimers(PBD)	DNA damaging agent	Recognize specific sequences of DNA, High antitumor activity	Cardiotoxicity	(69)
Duocarmycins	DNA damaging agent	Less drug resistance	More sensitive to PH changing	(70, 71)
Anthracyclines	DNA damaging agent	Powerful antiproliferative activity	Cardiotoxicity	(72)
Camptothecin analogues (SN-38, Dxd)	DNA topoisomerase I inhibitor	Low to medium toxicity, better bystander faster metabolism	Unstable in light poor water solubility, serious side effects	(66, 73, 74)
Amatoxin	DNA transcription inhibitor	Good water solubility		(16)
Thailanstatin A	Block RNA splicing	Powerful antiproliferative activity Targeting active dividing and resting cells	Lack of suitable bonding groups	(16)
Tetrahydroisoquinoline-fused benzodiazepine (TBD)	Lysosome-cleavable site-specific conjugation	High target specificity robust antitumor activity	Secondary processing results in cumbersome procedures	(75)
Silica nanoparticle	Enhance permeability and retention (EPR)	Superior specific cytotoxicity Multivalent binding property	Less safety	(76)
Eribulin	Microtubule inhibitor	High efficiency higher expression	High off-target toxicity	(77)
Cyclopropa [c] pyrrolo[3,2-e] indole-4-one dimer (CPI dimer)	DNA-damaging, enable DAR of 2	Safer		(78)
Alpha-Amanitin	Highly selective inhibition of RNA polymerase II	Small molecular weight Good water solubility	Sole source	(65)
proper concentration of nitric oxide(NO)	Promote tumor apoptosis through impacts on mitochondrial membrane permeability and discharge of cytochrome c oxidase	The effect of promoting tumor cells apoptosis is significant	Gas form limits building stable NO donors	(62)

### 3.2 Performance optimization

#### 3.2.1 Overcoming the multi-drug resistance of ADC

As the clinical applications of ADC are unfolding, it can be found that multi-drug resistance (MDR) has become a major factor limiting the effectiveness of clinical treatment and overall survival of patients, which makes it urgent to overcome the MDR of ADC to promote PK/PD. First of all, as mentioned above, drug resistance can be reduced by developing targeted small molecule toxins, reducing the hydrophobicity of linkers, or employing non-cleavable linkers as these approaches (56, 79).

In addition, recent studies have found that the production of MDR in ADCs is associated with the active pumping of cytotoxic agents out from the cell by the MDR1 glycoprotein in the tumor cell membrane. The modification of the linker by attaching maytansine (DM1) to the antibody using maleimide-based hydrophilic linker PEG4Mal can make the molecule released after ADC hydrolysis by intracellular enzymes a poor substrate for MDR1, thus avoiding the toxin being pumped out and overcoming drug resistance to improve PK/PD. Reducing the degradation and recycling of the target is also an effective approach to decreasing MDR. For example, the addition of HSP90 inhibitors reduces the degradation of HER2 and improves the circulation of HER2 *in vivo (* 80).

Furthermore, a study by Yamazaki et al. found that homogeneous dual-drug ADCs (incorporating two distinct payload molecules into single monoclonal antibodies using multi-loading linkers) have better resistance than coadministration of two single-drug ADCs carrying the same payloads, showing significant advantages in treating tumor cells with low expression of the target (81).

#### 3.2.2 Improving the tumor penetration of ADC

Apart from optimizing linkers, antibodies, antigens, and payload during development, maximizing the tumor penetration of ADC may contribute to the combination of drugs and target cells and the efficacy of the future in the clinic (82). One of the reasons for the low penetration of ADC in solid tumors may be due to binding-site barrier(BSB) (83), resulting in the distribution of mAb within solid tumors exhibiting a high degree of concentration heterogeneity (25, 84). Consequently, it is necessary to promote a more uniform distribution of ADC near solid tumors and thus improve the effective tumor penetration of ADC.

Designing antibody co-administration is one of the effective methods to promote uniform drug distribution and overcome BSB. A series of findings and mathematical modeling based on the PK/PD by Cornelius Cilliers et al. showed that co-administering trastuzumab with a fixed dose of T-DM1 at 3:1 and 8:1 ratio allowed for more uniform distribution of the drug within the tumor, significantly improved tumor penetration of ADC and increased median survival by twofold compared to T-DM1 alone (0:1). And this effect could be enhanced with the increase of the ratio in a certain range (0:1<3:1<8:1) (23, 82). Notably, however, subsequent studies have shown that it exhibits more significant inhibition of BSB under conditions of insignificant bystander effects, high antigen expression, and high dose administration. Conversely, it may fail to achieve significant efficacy improvement (84).

Additionally, the use of antigen-binding site competitive inhibitors is another example of the application of antibody co-administration. Bordeau and colleagues achieved transient inhibition of the high-affinity binding of trastuzumab antibodies to the antigen-binding site HER2 by adding the antigen-binding site competitive inhibitor 1HE (83). A combination of ImageJ and an in-house MATLAB algorithm showed that co-administration of 1HE with trastuzumab resulted in a significant increase in the proportion of trastuzumab-stained positive tumor sections [26.52% (SD, 8.11) - 43.32% (SD, 11.42)], improving drug distribution uniformity, thus significantly increasing penetration depth. The study showed that the upper percentage of tumor penetration distance was approximately 30% higher in the co-administered group than in the separately administered group (24). Studies have confirmed the significant value of antibody co-administration in improving drug killing of tumor cells.

#### 3.2.3 Surface modification of ADC

Since the pure component structure design of ADC has some performance defects inevitably, to fully utilize the efficacy of ADC and expand the therapeutic window, appropriate surface modifications are usually required to improve the overall performance of ADC. Glycosylation is a commonly used post-translational modification technique to selectively change the properties and effects of ADC by adding a certain number of glycan molecules to the side chain residues of antibodies and proteins in a targeted manner. On the one hand, the immune response can be potentiated or dampened by modulating the glycan composition and binding sites of antibody glycosylation modifications; on the other hand, Fc glycosylation modification can also enhance the thermodynamic and serum stability of antibodies, reduce plasma clearance and prolong the half-life of antibodies. As a result of these improved properties of antibodies, the duration of drug action is also extended (54). Besides, the pharmacokinetics of ADC can be optimized through PEGylation by adding a non-immunogenic polyethylene glycol (30) polymer to the molecule to improve certain performance defects of the target molecule, such as reducing drug immunogenicity, improving solubility, and half-life (85), regulating non-specific biodistribution and releasing peak tissue concentrations of payload (86). By increasing the volume and flexibility of the molecule, pegylation reduces the kidney’s clearance of the drug, while reducing the immune system’s recognition of the drug and reducing the immune response. In addition, pegylation is also able to improve the water solubility of the drug and reduce aggregation, which reduces side effects and improves efficacy. Through these mechanisms, pegylation helps to improve the efficacy and safety of ADCs, making them more effective in the treatment process.

#### 3.2.4 Exploiting the bystander effect of ADC

The non-selective and equal killing of antigen-positive(Ag(+)) and antigen-negative (Ag(-)) cells using the “bystander effect” of the drug is also one of the optimization strategies (87, 88). From one side, the “bystander effect” can make the payload more uniformly distributed in solid tumors and improve the heterogeneity of antigen expression on the surface of target cells. From the other side, the “bystander effect” may cause indiscriminate toxic effects on normal cells in the vicinity of target cells, resulting in more serious drug side effects.

In fact, only cleavable linker-mediated payload can usually produce bystander killing (89). This is due to the fact that only the neutral payload can be released from Ag(+) cells to achieve diffusion, while non-cleaved linkers always fail to meet this requirement (90). Therefore, linkers with different cleavage characteristics can be used to design bystander killing levels that meet the requirements. Additionally, the results of computational transport analysis by Eshita Khera and Cornelius Cilliers show that the lipophilicity of the payload affects the bystander effect dramatically, with low lipophilic load exhibiting long-range (hundreds of microns) and more rapid diffusion, while high lipophilic payload diffuses at a much lower rate and distance (tens of microns) (91). The lipophilicity level of the payload design can also effectively influence the strength of the bystander effect and thus expand the scope of action of ADC.

## 4 Optimization model and novel ADC based on PK/PD

Given the diversity and complexity of ADC research, the utilization of PK/PD-based mathematical models in the ADC design process enables quantitative assessment of parameters to determine appropriate drug structures, properties, doses, and administration routes. This approach provides a more comprehensive and precise framework for designing novel ADCs.

### 4.1 Modeling based on PK/PD

The complex structure of antibody-drug couplings poses a unique challenge to PK and PD characterization because it requires quantitative understanding of the PK and PD properties of many different molecular classes (e.g., couplings, total antibodies, and uncoupled payloads) in different tissues. Quantitative clinical pharmacology using mathematical modeling and simulation provides an excellent way to overcome these challenges, as it can integrate the PK and PD of the ADC and its components in a quantitative manner simultaneously.

#### 4.1.1 Drug structure

ADCs have complex molecular structures that combine the molecular properties of small-molecule drugs and large-molecule biotherapeutics. Conjugations of cytotoxic drugs with mAbs often result in a heterogeneous mix of antibody-drug conjugator species that differ not only in the number of cytotoxic drugs attached to the antibody (i.e., the drug-antibody ratio of species), but also in the different attachment locations on the antibody (92). PK/PD analysis and exposure response analysis were performed for safety-related analytes, such as specific adverse events of particular concern, adverse events occurring in≥grade 3 therapy, and drug structure adjustments due to safety adverse events (AEs) and efficacy response rates, and logistic regression was used to evaluate exposure measures (e.g., AUC, Cmax) and the endpoints and effects of covariates. Ruud Ubink et al. (93) found that carboxyleesterase 1c (CES1c) can cut the connector drug on the ADC at different sites, often resulting in the instability of the connector drug and poor PK/PD of several ADCs. Xenotransplantation studies of VC-Seco-DuBa-based ADCs, including SYD985, in mice with immunocompromised CES1c expression, have shown that PK/PD studies confirm the cleavage of VC-Seco-DuBa leads to covalent bond formation between CES1c and the ADC. This provides a preferred alternative for optimizing the structure of the conjugate drug.

#### 4.1.2 Property

New methods of property analysis are needed for ADCs to complement those used by the antibodies themselves. Adcs offer a number of physicochemical properties due to the conjugations themselves as well as the hydrophobic payloads that must be considered during their CMC development. The coupling of the hydrophobic payload to the mAb can form aggregates by increasing the hydrophobicity of the mAb. Drug coupling also disrupts local secondary and tertiary structures, resulting in adverse conformational changes. The same is true of the development of novel linkers and payloads and their impact on the ADC’s structural and functional properties, including specificity, toxicity, solubility, and stability. Barbara Valsasina et al. (94) optimized the toxin to choose the best splicer to balance reactivity and stability. In the HER2-driven model, A murine-based PK/PD model predicted tumor regression in patients after administration of 2 doses of trastuzumab - NMS-P945 - ADC at 0.5mg/kg, and observed high *in vivo* efficacy in cured mice at a well-tolerated dose. A novel trastuzumab - NMS-P945 - ADC suitable for coupling with monoclonal antibodies with DAR>3.5 was developed. The ADCs it produces have good internalization properties, are capable of inducing bystander effects and immunogenic cell death, and represent a highly efficient innovative payload for creating new next-generation ADCs.

#### 4.1.3 Dosage

For small - or large-molecule anticancer drugs, the transition of efficacy from the laboratory to the bedside has been challenging. Use established *in vitro* and preclinical experimental systems to build confidence in drug conversion and use mathematical models to interpret these data to predict the clinical efficacy of the drug. PK/PD model can characterize the *in vitro* bystander effect of ADC in heterogeneous tumors. There are currently five ADCs and > 80 molecules in clinical development, but their efficacy is often limited by poor tumor distribution and the heterogeneity of antigen-expressing cells (95). These limitations can be overcome with the help of bystander effects, but to date, the incidence and extent of bystander effects in heterogeneous tumors have not been quantitatively determined or mathematically characterized (88). The heterogeneous tumor PK model was integrated with the PD model, which used the intracellular occupancy of tubulin as the driver of ADC efficacy. The final model was able to reasonably characterize all tumor growth inhibition (TGI) data simultaneously using a set of PD (Kmax, KC50, γ) parameters. Aman P Singh et al. (84) used a semi-mechanical PK-PD model to quantitatively characterize TGI data, and simulated the effects of different drug administration protocols and tumor components on ADC bystander effects by establishing PK-PD relationships between tumor drug concentrations and TGI data obtained from different xenografts. Model simulations suggest that dose grading may further improve the overall efficacy and bystander effect of ADCs by extending tubulin occupancy in each cell type.

#### 4.1.4 Administration route

Different routes of administration may lead to changes in PK/PD behavior of ADCs and may help improve their therapeutic index. To evaluate this hypothesis, Chang et al. (96) evaluated PK/PD for ADCs administered via intravenous (IV), subcutaneous (SC), and intratumoral (IT) pathways, and developed a cyborg PK/PD model to characterize both PK and TGI data for ADCs. The PK curves of total mAb, total monomethyl auristatin E (MMAE), and uncoupled MMAE in plasma and tumor after T-vc-MMAE administration in IT, SC, and IV were fitted with the model, and the model predicted a single tumor growth curve was superimposed on the PD data of observed IT, IV, and SC administration routes. The results showed that IT administration of ADC significantly increased tumor ADC exposure and enhanced anti-tumor activity *in vivo*. In addition, model simulations suggest that IT injections can potentially improve the therapeutic index of ADC compared to traditional IV injections, on the other hand, SC therapy is less effective *in vivo* than IV therapy.

### 4.2 Novel form of ADC

A pivotal consideration in the design of novel ADCs is the optimization of PK/PD, aimed at enhancing therapeutic efficacy through improved *in vivo* drug behavior. Next-generation ADCs are at the forefront of targeted oncology, integrating cutting-edge technologies to enhance PK/PD profiles. Through meticulous engineering of the ADC’s constituent elements—antibody, linker, and payload—these innovative therapies are designed to achieve superior targeting precision and mitigate adverse effects.

#### 4.2.1 Non-internalizing ADC

Non-internalizing antibody-drug conjugates (ADCs) represent a novel paradigm in targeted therapeutics (97). In contrast to traditional ADCs that depend on internalizing receptors to achieve the release mechanism of the payload within the cell, these ADCs target non-internalizing receptors or extracellular matrix proteins and use bioorthogonal chemical reactions to selectively cut the link between the antibody and the payload *in vivo* by administering a linker reactivator later on. This allows the release of the drug and its uptake by surrounding cancer cells and tumor supportive stromal cells, ultimately leading to tumor cell apoptosis (98). The fundamental principle is rooted in the Diels-Alder (IEDDA) conjugate of reverse electron demand (99), wherein the interaction between trans-cyclooctene (TCO) and butyrazine derivatives and the cleavage of allyl carbamate from TCO during the reaction with tetrazine (100) realize the “Click-to-Release” effect of ADC in the extracellular environment. This category of ADC not only transcends the conventional limitations of targeting exclusively internalizing receptors, thereby expanding the spectrum of actionable targets, but also facilitates the precise modulation of the drug release dynamics, allowing for a more controlled and targeted therapeutic intervention. In addition, the extracellular release of therapeutics promotes drug diffusion to adjacent tumors, thereby enhancing the bystander effect, especially improving the efficacy for solid tumors with poor penetration and specificity (98).

#### 4.2.2 Dual-site and dual-targeted ADC

Bispecific ADCs (BsADCs), recognized by the academic community as a next-generation targeted drug design strategy, can be categorized into two types based on their binding modes: dual-site ADCs and dual-targeted ADCs (101, 102). As the name implies, dual-site are ADCs that target different sites on the same antigen.; dual targets are ADCs that target two different antigens. In comparison to mAb, BsAbs have lower off-target toxicity, more rapid internalization, and lower drug resistance (54, 103). Kast and colleagues designed anti-human epidermal growth factor receptor 2 (HER2) Biparatopic ADCs and quadrivalent IgG fusion ADCs with multimodal mechanisms of action, both based on a dual-epitope framework. The geometry of the ADC binding domain was identified as critical for HER2 antagonism, with some configurations exhibiting agonist activity (104). Currently, 10 BsADCs are undergoing clinical trials. Despite the remarkable research outcomes achieved thus far, their safety and efficacy still do not align with theoretical expectations (101). it remains essential to optimize them based on pharmacokinetics, and conduct further basic and clinical studies to achieve systematic improvement.

#### 4.2.3 Dual-drug ADC

Dual-drug ADC is the conjunction of two different loads or linkers on the same antibody to addressing the common drug resistance problem of continuous administration (60). Dual-drug ADC can be categorized into single site and double site schemes according to the mode of loading and antibody attachment. The former can be subdivided into series and parallel forms, and its technical difficulty is relatively low, garnering more attention in recent literature. Tang et al. successfully developed semi-site-specific dual-drug ADCs by combining the one-pot chemoenzymatic synthesis of glycoengineered herceptin with novel non-natural egg-yolk sialyl glycopeptide (SGP) carrying azido or alkyne tags (105). Furthermore, the researchers have developed a multi-plexing drug that is conjugated to native antibody interchain disulfides by a stable maleimide chemistry carrier aimed at minimizing the dissociation of drug connectors *in vivo*. The vector contains two orthogonally protected cysteine residues that can be conjugated to distinct drug linkers, allowing multiple drugs to be evenly distributed between the two drug linkers while preventing ADC aggregation effects due to hydrophobicity (106). Research has demonstrated that homogeneous ADCs incorporating two distinct payloads are a promising therapeutic strategy to combat the heterogeneity and drug resistance associated with breast cancer. The researchers efficiently synthesized conjugates with defined DARs with combined DARs of 2 + 2, 4 + 2, and 2 + 4. These constructs exhibit HER2-specific cytotoxicity at therapeutic doses, desirable pharmacokinetic profile, less inflammatory response, and off-target toxicity (81). The double site employs a more intricate conjunction mechanism to associate distinct payloads at various antibody sites. For example, Nilchan et al. reported their design of a dual-drug ADC that targets HER2 and couples Virtual DNA crosslinking agent PNU-159682 and tubulin polymerization inhibitor monomethyl auristatin F (MMAF) via engineered site-specific (Sce) and cysteine (Cys). A series of *in vitro* studies have validated its dual mechanistic characteristics (107).

#### 4.2.4 Peptide drug conjugate (PDC)

The Peptide drug conjugate (PDC) consists of three components: targeted peptide, payload and linker. Structurally akin to ADC, PDCs utilize peptides with lower molecular weights as targeted delivery vehicles to enhance targeting efficiency and facilitate drug penetration (108). Based on their functional characteristics, targeted peptides can be categorized into cell-penetrating peptides, cell-targeting peptides, self-assembling peptides (SAPs), and responsive peptides (109, 110). Cell-penetrating peptides are classified into linear and cyclic peptides in structure, typically comprising 5 to 30 amino acids. Due to the small and compact structure, can traverse cellular membranes without compromising membrane integrity, thereby facilitating penetrating the blood-brain, blood-eye and other physiological barrier (110). This property enables their application in treating diseases such as the brain, central nervous system, and other regions of the body. Cell-targeting peptides are defined as peptides showing cell- or tissue-specific binding activity, the prevalent types include arginine-glycine-aspartate (RGD) peptide, gonadotropin-releasing hormone (GnRH) peptides and somatostatin (SST) mimetic peptides (111). SAPs and responsive peptides have increasingly garnered the attention of researchers due to their respective characteristics of biocompatibility and environmental responsiveness (110). The pharmacokinetic properties of PDC, including insufficient stability, uneven distribution, and a short circulation time, serve as critical guidelines for developing PDC with enhanced efficacy (112). A primary challenge in its development is addressing the short circulating half-life and off-target side effects (108). Enhancing peptide specificity and safety through cyclization, nanoparticles, natural amino acid modifications, and computer-assisted technology such as molecular dynamics simulations and molecular docking has become a focal point in contemporary PDC research (112).

#### 4.2.5 Probody–drug conjugates

Probody-drug conjugates is an innovative antibody engineering strategy designed to improve the targeting of antibody drugs and reduce side effects. It can be categorized into protease-sensitive self-masking moieties and pH-responsive antigen-binding sites according to their mechanism of action. The former elicits specific anticancer activity through cleavable a peptide sequence (LSGRSDNH) that has minimal activity in healthy tissues but is significantly upregulated in malignant ones. The latter accomplishes this pH-dependent activation mechanism by integrating weakly basic histidine residues into the antibody’s binding regions (60). The core principle of probody-drug conjugates is to attach a masking peptide to the target binding region of antibodies, effectively concealing the active site and thereby restricting antibody activation in normal tissues, which significantly mitigates off-target toxicity associated with these drugs (113, 114). Boustany et al. presented CI107, a probody T cell-engaging bispecific antibodies targeting EGFR and CD3. *In vivo* experiments demonstrated that compared to unmasked CI107, the cytotoxic activity of dual-masked CI107 was diminished by over 15,000-fold. Similar findings were corroborated through additional *in vivo* experiments (115). Trang and colleagues proposed an innovative antibody design wherein a heterodimeric coiled-coil domain masks the binding domain of the antibody. The curly helix peptide will only cleave and resume antigen binding upon exposure to tumor-associated proteases, thereby substantially enhancing the drug’s circulating half-life (116).

## 5 Conclusion and future perspectives

As mentioned above, we’ve expounded comprehensively on applying pharmacokinetics or pharmacodynamics while designing ADC to obtain higher efficacy and fewer side effects. From the ADC’s PK/PD property while coming into play *in vivo* and the PK/PD study strategy, to specific ADC optimization methods and recommendations based on PK/PD, it has been study-approved that the PK/PD properties exert a subtle role in the development of ADC, whether in preclinical trials or clinical promotion. The study of PK/PD unfolds the detailed mechanism of ADC action, making it easier to control related parameters in the process of designing ADC. In the ADC design process, the PK/PD-based mathematical model can be used to evaluate the parameters quantitatively, which provides a more comprehensive and accurate framework for designing new ADC. However, limited efficacy and inevitable off-target toxicity remain a challenging bottleneck, especially when binding with the complex PK characteristics and individual patient specificity.

The limitation of this article primarily resided in the insufficient consideration of the influence of the internal environment on the pharmacokinetics (PK) and pharmacodynamics (PD) of antibody-drug conjugates (ADCs) within the body, along with the potential impact of interactions with other food or beverage components on these PK/PD processes. However, we focused more on the process of ADCs alone in the body. In our future research, we will focus on studying the impact of DDI or certain body components or functional states on PK/PD process, which will guide us to explore the optimal internal environment for ADCs to achieve better drug efficacy, with relatively appropriate PK/PD parameters. Based on the PK/PD model, meanwhile in combination with specific internal environment control, safer and more effective ADCs will be born. The development of ADCs is facing a multi-aspects challenge. Currently, numerous studies are overcoming the barrier, trying to upgrade ADC into a more profitable therapy for patients, which requires considering several related factors. This may include an overall estimation of the PK/PD property while the ADC function, and the appropriate strategy maximize the effect. Looking ahead, ADCs may undergo a significant transformation, evolving into immunotherapy agents that harness the cytotoxic potential of PD-1/PD-L1 or CTLA-4 immunotherapies through the optimization of ADC payload’s PK/PD properties. Thus, the field of ADC development can consider have been expanded, and not have been restricted to typical directions. In addition, by fully grasping ADC’s PK/PD properties and making the utmost of it in the process of optimizing ADC, the development of precise therapy for cancer can develop farther away.
